# An in vitro study of ApxI from *Actinobacillus pleuropneumoniae* serotype 10 and induction of NLRP3 inflammasome‐dependent cell death

**DOI:** 10.1002/vro2.20

**Published:** 2021-10-04

**Authors:** Eduardo Hernandez‐Cuellar, Alma Lilián Guerrero‐Barrera, Francisco Javier Avelar‐Gonzalez, Juan Manuel Díaz, Jesús Chávez‐Reyes, Alfredo Salazar de Santiago

**Affiliations:** ^1^ Laboratorio de Biología Celular y Tisular Departamento de Morfología Universidad Autónoma de Aguascalientes (UAA) Aguascalientes Mexico; ^2^ Laboratorio de Ciencias Ambientales Departamento de Fisiología y Farmacología Universidad Autónoma de Aguascalientes (UAA) Aguascalientes Mexico; ^3^ Facultad de Medicina e Ingeniería en Sistemas Computacionales de Matamoros Universidad Autónoma de Tamaulipas Tamaulipas México; ^4^ Unidad Académica de Odontología Área de Ciencias de la Salud Universidad Autónoma de Zacatecas Zacatecas México; ^5^ Unidad Médico Didáctica Centro de Ciencias de la Salud Universidad Autónoma de Aguascalientes Aguascalientes México

## Abstract

**Background:**

*Actinobacillus pleuropneumoniae* (AP) is the causative agent of porcine pleuropneumonia. Apx exotoxins are the most important virulence factors associated with the induction of lesions. ApxI is highly cytotoxic on a wide range of cells. Besides the induction of necrosis and apoptosis of ApxI on porcine alveolar macrophages (PAMs), its role in pyroptosis, a caspase‐1‐dependent form of cell death, has not been reported. The aim of this study was to analyse if NLRP3 inflammasome participates in cell death induced by ApxI.

**Methods:**

PAMs, the porcine alveolar macrophage cell line 3D4/21 and a porcine aortic endothelial cell line were used in this study. We used Z‐VAD‐FMK and Ac‐YVAD‐cmk to inhibit caspase‐1. Glyburide and MCC950 were used to inhibit the NLRP3 inflammasome. A lactate dehydrogenase release assay was used to measure the percentage of cell death. Caspase‐1 expression was analysed by immunofluorescence. End‐point RT‐PCR was used to analyse the expression of NLRP3 mRNA.

**Results:**

Rapid cell death in PAMs, 3D4/21 cells and the endothelial cell line were induced by ApxI. This cell death decreased by using caspase‐1 and NLRP3 inflammasome inhibitors and by blocking the K^+^ efflux. Expression of NLRP3 mRNA was induced by ApxI in alveolar macrophages while it was constitutive in the endothelial cell line. Detection of caspase‐1 in alveolar macrophages was higher after ApxI treatment and it was blocked by MCC950 or heat inactivation.

**Conclusions:**

To the best of the authors' knowledge, we have described for the first time in vitro induction of ApxI associated pyroptosis in alveolar macrophages and endothelial cells, a rapid cell death that depends on the activation of caspase‐1 via the NLRP3 inflammasome.

## INTRODUCTION

*Actinobacillus pleuropneumoniae* (AP) is a Gram‐negative rod and the aetiological agent of the porcine pleuropneumonia, a highly contagious respiratory disease produced in pigs of all ages with a worldwide distribution. The disease is characterised by fibrinohaemorrhagic necrotising pleuropneumonia that leads to sudden death in the acute infection with economic losses to the swine industry. There is a classification of AP based on the requirement of the cofactor nicotinamide adenine dinucleotide (NAD) to grow in vitro. Biotype 1 strains are NAD‐dependent while biotype 2 strains are NAD‐independent. There are 18 serotypes of AP based on the antigenic properties of their capsular polysaccharides.^1^, ^2^

One of the most important virulence factors associated with the induction of lesions by AP is the production of the pore‐forming exotoxins ApxI, ApxII and ApxIII. These toxins are members of the repeats in toxin family and have several degrees of haemolytic and cytotoxic activity.^3^ Depending on the serotype of AP, different combinations of Apx exotoxins are expressed. Serotypes 1, 5, 9 and 11 produce ApxI and ApxII. Serotypes 2, 3, 4, 6, 8 and 15 produce ApxII and ApxIII. Serotypes 7, 12 and 13 generate only ApxII, while serotypes 10 and 14 generate only ApxI.^4^ ApxI was shown to possess cytotoxic activity on alveolar macrophages and neutrophils.^1^, ^5^ Also, ApxI was described to induce apoptosis in porcine alveolar macrophages (PAMs) via caspase‐3,^4^ caspase‐8 and caspase‐9. It was also found that the mitogen‐activated protein kinases p38 and JNK participated in that signalling pathway.^6^

Besides the description of the necrotic lesions and the induction of apoptosis in PAMs by ApxI toxin or AP,^7^ there are no reports of other types of cell death including pyroptosis, a caspase‐1‐dependent form of cell death. It has been shown that several pore‐forming toxins induce the activation of the NLRP3 inflammasome, a molecular platform that activates caspase‐1 with the consequent processing of pro‐IL‐1β and pro‐IL‐18, producing the release of the active forms of these cytokines during pyroptosis.^8^, ^9^, ^10^ Furthermore, it was demonstrated that the efflux of K^+^ is the mechanism of activation of the NLRP3 inflammasome by pore‐forming toxins.^11^ Because of the haemorrhagic process during the acute infection with AP and the inflammatory response associated with generalised respiratory distress in porcine pleuropneumonia, we decided to analyse in vitro the activation of the NLRP3 inflammasome in alveolar macrophages and endothelial cells.

## MATERIALS AND METHODS

### Cell lines, bacterial strains and reagents

The porcine alveolar macrophage cell line 3D4/21 (ATCC CRL‐2843) and a porcine aortic endothelial cell line previously reported ^12^ were used in this study. Cells were cultivated in RPMI‐1640 media (Sigma, #R8755) supplemented with 10% fetal bovine serum (FBS; Gibco, 16000044) and a mixture of antibiotics containing 10 U/ml of penicillin, 10 mg/ml of streptomycin (Gibco, #15140122), 100 mg/L of gentamicin (Gibco, #15750060) and 2.5 mg/ml of amphotericin B (Gibco, #15290018). Cells were incubated at 37^o^C in 5% of CO_2_. For the experiments, around 2.5 × 10^5^ cells per well were seeded into 48‐well plates. AP serotype 10 was donated by Mario Jacques (Faculté de Médecine Vétérinaire, Université de Montréal, Canada). Bacteria were cultured in brain heart infusion broth (BHI; BDBioxon, #211200) or agar (BDBioxon, #214700) supplemented with 10 μg/ml of NAD (Affymetrix, #53849) at 37°C.

For the inhibition of caspase‐1, the inhibitors Z‐VAD‐FMK (InvivoGen, #tlrl‐vad) and Ac‐YVAD‐cmk (InvivoGen, #inh‐yvad) were used. For inhibition of NLRP3, Glyburide (Sigma, #G0639), MCC950_1 (TOCRIS, #5479) and MCC950_2 (Sigma, #PZ0280) were used 30 min before the addition of ApxI. To remove the effect of contaminating lipopolysaccharide (LPS) in the whole toxin extract the antibiotic polymyxin B (InvivoGen, #tlrl‐pmb) was used at the same time as the ApxI treatment. To inhibit the potassium efflux, KCl (Potassium chloride; J. T. Baker, #3040‐01) was added at the same time as the ApxI treatment.

### Isolation and culture of PAMs

Isolation of PAMs was performed as previously reported with minor modifications.^13^ Briefly, pigs from 2–14 weeks of age were sacrificed following an animal protocol approved by the animal ethics committee of the Autonomous University of Aguascalientes, México. Then, instillation of the lungs with phosphate‐buffered saline (PBS) containing 10 units/ml of penicillin, 10 μg/ml of streptomycin, 100 mg/L of gentamicin and 250 g/L of amphotericin B was carried out. The PBS suspension was centrifuged at 95 g and the pellet containing PAMs was washed three times with PBS containing antibiotics. In the last wash, the cells were replaced with a culture medium or freezing medium (culture medium adjusted at 20% FBS plus dimethyl sulfoxide 10%) in case of freezing in liquid nitrogen. For experiments, cells were incubated at 37^o^ C in 5% of CO_2_.

### ApxI toxin extraction from AP serotype 10

Preparation of ApxI from AP serotype 10, which expresses and secretes only ApxI, was performed as previously described with minor modifications.^4,^ ^14^ Briefly, bacteria were incubated on BHI agar containing 10 μg/ml of NAD for 24 h at 37°C. The next day, some colonies were inoculated into BHI broth supplemented with 10 μg/ml of NAD and cultured for 5 h at 37°C while shaking at 50 RPM. The bacterial suspension was centrifuged at 16,000 g for 10 min at 4°C and the resulting pellet was replaced with RPMI‐1640 supplemented with 5% FBS. This new bacterial suspension was cultured for two more hours at 37°C while shaking at 50 RPM. Finally, the culture was centrifuged and the supernatant containing the exotoxin was filtered through 0.22 μm filter, aliquoted and frozen at −70^o^ C for further experiments. The concentration of proteins in the whole extract containing the toxin was around 1 mg/ml measured by the Bradford protein assay. The concentration of the extract containing ApxI used for experiments was based on the cytotoxicity in PAMs, with dilution 1/4 having around 50% of cell death assessed by the lactate dehydrogenase (LDH) release assay.

### Cytotoxicity of cells treated with ApxI

PAMs, alveolar macrophages 3D4/21 and the endothelial cells were treated with ApxI. Then the supernatant was collected to determine the cytotoxic effect by the release of the enzyme LDH using the LDH‐Cytotoxicity Assay Kit II (Biovision, #K313) according to the manufacturer's instructions. The absorbance was measured at an optical density of 495 nm using a microplate reader.

### Evaluation of NLRP3 expression by RT‐PCR

Porcine alveolar macrophages, 3D4/21 and endothelial cells were treated with ApxI for 1 h. Then the supernatant was removed and cells were lysed with TRIzol reagent (Ambion, #15596026). Direct‐zol RNA Miniprep (Zymo Research, #R2050) was used to purify total RNA following the manufacturer's instructions; the step of DNaseI treatment was included. For cDNA synthesis using the oligo (dT)_18_ primer, we used the RevertAid First Strand cDNA synthesis kit (Thermo Scientific, #k1622). Finally, PCR was carried out using *Taq* DNA Polymerase with Standard *Taq* Buffer (New England Biolabs, #M0273). The following primers were used: (Housekeeping) GAPDH sense: 5′‐GGCTGCCCAGAACATCATCC‐3′, antisense: 5′‐GACGCCTGCTTCACCACCTTCTTG‐3′ and NLRP3 sense: 5′‐GTGGTAACAGCCTGGGAGAC‐3′, antisense: 5′‐GTGCAACAGTCCCTCACAGA‐3′. After the agar gel electrophoresis, the GAPDH transcript resulted in a band with a molecular size of around 195 bp while the NLRP3 transcript was around 569 bp as expected.

### Detection of caspase‐1 by immunofluorescence

PAM 3D4/21 cells were incubated with or without the NLRP3 inflammasome inhibitors 30 min before ApxI exposure for 1 h. Then, cells were fixed with 3.7% formaldehyde. After washing with PBS, cells were permeabilised with 0.1% Triton X‐100 for 5 min and then incubated with blocking solution, PBS with 5% bovine serum albumin for 30 min. To label caspase‐1, a primary mouse monoclonal IgG_1_ anti‐Caspase‐1 antibody was used (Santa Cruz Biotechnology, #14F468), incubating for 2 h at 37°C. This was followed by incubation with the secondary polyclonal anti‐mouse antibody conjugated with Alexa Fluor 488 (Sigma, #SAB4600042). Finally, the samples were nuclear counterstained with Hoechst 33342 (Thermo Scientific, #62249) and mounted with ProLong Gold antifade reagent (Invitrogen, #P36930). Images were captured with an inverted confocal microscope (Zeiss, LSM700) and processed with the software ZEN Black 2.3 SP1.

### Statistical methods

GraphPad Prism 8.0 was the software used to evaluate the quantitative data in this study. We used one‐way ANOVA with multiple comparisons. Dunnett's test to compare with a control group or Tukey's test to compare all the groups among them.

## RESULTS

### ApxI induced cell death in PAMs

**FIGURE 1:**
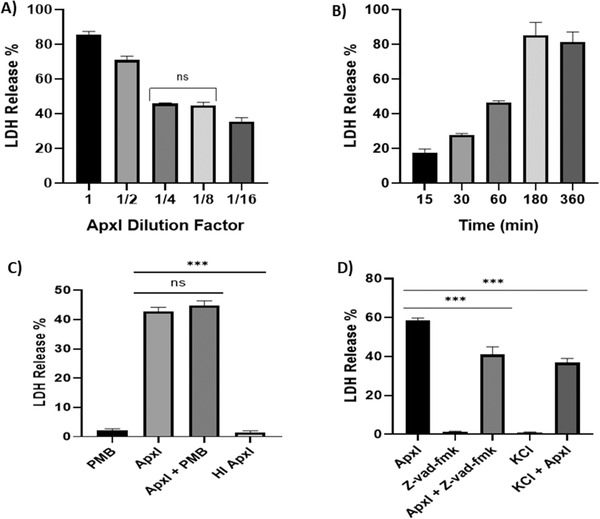
ApxI induced cell death in porcine alveolar macrophages (PAMs) is inhibited by Z‐VAD‐FMK and potassium chloride (KCl). (a) PAMs were treated with the whole ApxI extract or 2‐fold dilutions for 1 h. Cytotoxicity was measured by lactate dehydrogenase (LDH) release assay. (b) Cytotoxicity of PAMs treated with ApxI at different time points. (c) Cytotoxicity of PAMs treated with ApxI in the presence or absence of polymyxin B (PMB) at 10 μM/ml or with the heat‐inactivated (HI) ApxI toxin. (d) Cytotoxic analysis with or without the pretreatment of PAMs with Z‐VAD‐FMK (20 μM) 30 min before ApxI or at the same with KCl (62.5 mM). The experiments were analysed statistically using one‐way ANOVA, Tukey's test (***p ≤ 0.05)

PAMs were treated with supernatant containing ApxI toxin or 2‐fold dilutions for 1 h. As shown in Figure 1a, there was cell death that increased in a concentration‐dependent manner. Using the concentration of toxin that produced around 50% of cell death (dilution 1/4) in the previous experiment, it was found that cell death in PAMs started, from 15 min after toxin challenge and increased in a time‐dependent manner (Figure 1b).

It was assumed that extraction of toxins belonging to the repeats in toxin family may lead to contamination with LPS and that heat inactivation is an appropriate control method to show the bioactivity of the toxins in a whole extract.^16^ Consequently, polymyxin B was used to exclude the effect of LPS from the extract containing ApxI. Treatment of PAMs with ApxI in the presence of polymyxin B did not alter the percentage of cell death. This suggested that any contaminating LPS in the toxin extract did not contribute to the cytotoxic effect. Furthermore, cell death was completely abrogated when the toxin was heat‐inactivated at 96°C for 30 min, suggesting that the bioactivity of the toxin lost by heat inactivation is responsible for the cytotoxicity (Figure 1c).

### Inhibition of caspase‐1 and NLRP3 and effect on the cell death induced by ApxI

In order to assess the mechanism of cell death in PAMs challenged with ApxI, we added Z‐VAD‐FMK or KCl (to block potassium efflux) to the cells prior to the ApxI treatment. In Figure 1d, it is shown that in both situations there was a similar decrease in the degree of cell death induced by the toxin at 1 h.

**FIGURE 2:**
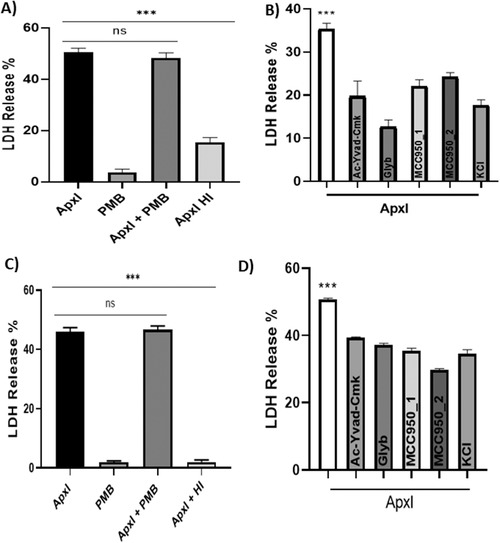
Caspase‐1 and NLRP3 inflammasome inhibitors and cell death induced by ApxI in porcine alveolar macrophage 3D4/21 and porcine aortic endothelial cell lines. (a) The porcine alveolar macrophage cell line 3D4/21 was treated with ApxI for 1 h in the presence or not of polymyxin B (PMB) (10 μM/ml) or with the heat‐inactivated ApxI. Cytotoxicity was measured by lactate dehydrogenase (LDH) release assay (***p ≤ 0.05; one‐way ANOVA, Tukey's test). (b) Cytotoxicity of the cell line 3D4/21 was measured at 1 h with or without the pretreatment for 30 min of Ac‐YVAD‐cmk (40 μM), MCC950_1 (10 μM), MCC950_2 (10 μM), Glyburide (20 μM) or at the same time of the ApxI toxin with KCl (62.5 mM) (***p ≤ 0.05; one‐way ANOVA, Dunnett's test). (c) The porcine endothelial cell line was treated with ApxI for 1 h in the presence or not of PMB (10 μM/ml) or with the heat‐inactivated ApxI (***p ≤ 0.05, one‐way ANOVA, Tukey's test). (d) Cytotoxicity of the endothelial cell line at 1 h with the inhibitors used in B before the addition of ApxI (***p ≤ 0.05; one‐way ANOVA, Dunnett's test). All the samples were tested by triplicate (*n* = 3) and the experiments were carried out three times. Ac‐YVAD‐cmk is an inhibitor of the Caspase‐1 enzyme; MCC950_1 and _2, glyburide are inhibitors of the NLRP inflammasome; KCl inhibits the K^+^ efflux pump that activates NLRP via the Apx1

Using the alveolar macrophage cell line 3D4/21 and a porcine endothelial cell line, we observed high levels of LDH release at 1 h of ApxI treatment (Figure 2a,c). Cell death was almost abrogated with heat inactivation of ApxI and polymyxin B did not alter the percentage of cell death. The caspase‐1 inhibitor Ac‐YVAD‐cmk and the NLRP3 inflammasome inhibitors Glyburide and two brands of MCC950 were tested with the alveolar macrophage and endothelial cell line. There was a significant decrease in the cell death of both cell lines when these inhibitors were used, indicating that caspase‐1 via the NLRP3 inflammasome was partially responsible for the total cell death generated by ApxI at this early time (Figure 2b,d). Furthermore, the addition of extracellular KCl to block the K^+^ efflux also decreased the cell death induced by ApxI in both cell lines.

### Expression of NLRP3 and caspase‐1 activity

**FIGURE 3:**
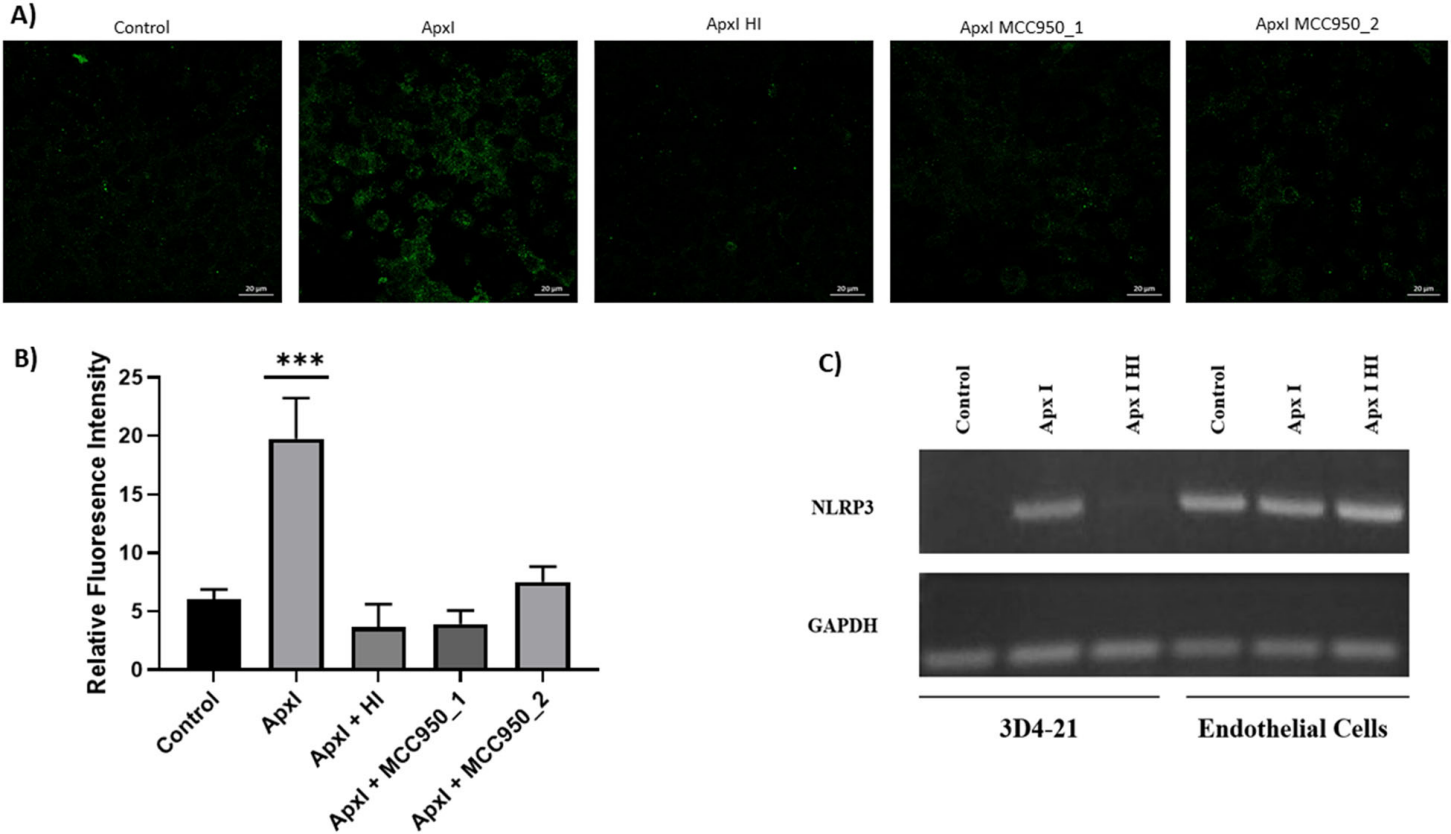
ApxI and expression of the NLRP3 transcript and caspase‐1 activation in alveolar macrophages. (a) The porcine alveolar macrophage cell line 3D4/21 was treated with ApxI for 1 h. Immunofluorescence of caspase‐1 using an antibody that detects the full‐length and the activated form of caspase‐1 was used. Heat inactivation or pretreatment for 30 min with MCC950 (10 μM) decreased the detection of caspase‐1. (b) Quantitative analysis of fluorescence intensity of caspase‐1 from the previous experiment with ImageJ software (***p ≤ 0.05; one‐way ANOVA, Tukey's test). (c) Total RNA extraction and endpoint RT‐PCR was carried out to detect GAPDH (house keeping) and NLRP3 mRNA transcripts by electrophoresis. Experiments were repeated three times with similar results

Following the challenge of the alveolar macrophages cell line with ApxI, we determined the presence of caspase‐1. Using confocal microscopy a fluorescent signal was apparent in cells treated with ApxI (Figure 3a,b). The signal remained similar to that in the control cells when cells were treated with MCC950 before the addition of the toxin or when the toxin was heat‐inactivated, suggesting that ApxI activated caspase‐1. ApxI was associated with the induction of mRNA NLRP3 transcript in alveolar macrophages; activity of the toxin was abrogated by heat inactivation (Figure 3c). In the endothelial cell line, the expression of the NLRP3 transcript was constant and independent of the treatment.

## DISCUSSION

The primary aim of this study was to evaluate the role of the NLRP3 inflammasome in the cell death induced by ApxI in PAMs and endothelial cells. The NLRP3 inflammasome is a multiprotein complex that promotes the activation of caspase‐1, inducing the processing and secretion of the immature proinflammatory cytokines IL‐1β and IL‐18 as well as pyroptosis.^15^ The NLRP3 inflammasome has been reported to be activated by several pore‐forming exotoxins^8^, ^9^, ^10^. We found that ApxI induced cell death in PAMs as early as 15 min and increasing in a time‐dependent manner. By comparison, ApxI has been reported to induce apoptosis in PAMs starting at 4 h after treatment with activation of caspase‐3 at 6 h.^3^, ^4^ This cell death was completely abrogated by heat inactivation of the toxin and the addition of polymyxin B to remove any contaminating LPS from the ApxI extract did not change the percentage of cell death, indicating that LPS did not contribute to this type of cell death. We used Z‐VAD‐FMK, an inhibitor of caspases that has been widely used to inhibit caspase‐1 via the NLRP3 inflammasome; we found a slight, but significant decrease in the total cell death induced by ApxI in PAMs. It has been shown that the mechanism through which the exotoxins induce the activation of the NLRP3 inflammasome is the efflux of K^+^.^11^ We found by blocking the K^+^ efflux by adding KCl to the medium at the same time as the ApxI treatment that a similar statistically significant decrease in the cell death as that produced with the caspase‐1 inhibitor.

We found that cell death induced by ApxI also occurred in the PAM 3D4/21 and porcine endothelial cell lines, with cytotoxicity evident in both cell lines at 1 h. In order to further characterise the mechanism by which this cell death occured, we tested several inhibitors of the NLRP3 inflammasome in both cell lines. Ac‐YVAD‐cmk is a cell‐permeable, selective and irreversible inhibitor of caspase‐1, when cells were treated with this inhibitor we found that there was a significant decrease in the total cell death induced by ApxI. Also, Glyburide and two brands of MCC950, inhibitors of NLRP3,^17^, ^18^, ^19^ were associated with a similar decrease of the total cell death induced by ApxI in both cell lines. Blocking the potassium efflux by adding KCl also resulted in a signifcant decrease in cell death. We suggest that besides necrosis as a consequence of the pore‐forming activity of ApxI in PAM cells, that an early caspase‐1‐dependent cell death also occurs via the NLRP3 inflammasome.

We observed that ApxI induced the expression of NLRP3 at the transcriptional level in alveolar macrophages and heat inactivation of the toxin eliminated that effect. In contrast, in the porcine endothelial cell line, the mRNA expression of NLRP3 was constitutive and unaffected by ApxI. It is well known that activation of the NLRP3 inflammasome starts through priming via TLR receptors or cytokine signalling with activation of the NFκB pathway in order to induce the mRNA expression of NLRP3.^20^ We suggest that ApxI induces the activation of the NFκB pathway independent of the TLR priming as it has been previously reported.^21^, ^22^ Furthermore, we found, using an antibody that recognizes the full‐length and the activated cleaved form of caspase‐1, that the detection of caspase‐1 was higher in alveolar macrophages challenged with ApxI. Heat inactivation or pretreatment with the NLRP3 inhibitor MCC950 decreased that level of detection, suggesting again that ApxI induces the activation of caspase‐1 via the NLRP3 inflammasome.

In conclusion, Apx exotoxins are probably the most relevant virulence factors in the infection with AP. We describe, to the best of our knowledge, for the first time that ApxI induces pyroptosis in alveolar macrophages and endothelial cells, a form of cell death that depends on the activation of caspase‐1 via the NLRP3 inflammasome. This may have clinical implications, given that caspase‐1 may be important in the cell death that can be associated with the haemorrhagic processes seen in porcine pleuropneumonia and also in the inflammatory responses associated with pneumonia, which is seen following the respiratory response in the acute phase of infection with AP.

## CONFLICT OF INTEREST

The authors declare that they have no conflict of interest.

## ETHICS APPROVAL

It was followed an animal protocol approved by the animal ethics committee of the Autonomous University of Aguascalientes, México.

## AUTHOR CONTRIBUTIONS

Eduardo Hernandez‐Cuellar designed the study. Juan Manuel Díaz and Alfredo Salazar de Santiago carried out the experiments. Eduardo Hernandez‐Cuellar, Juan Manuel Díaz and Jesús Chávez‐Reyes analysed the data. Eduardo Hernandez‐Cuellar wrote the original draft. Alma Lilián Guerrero‐Barrera, Francisco Javier Avelar Gonzalez and Jesús Chávez‐Reyes reviewed and edited the original draft. All authors have read and approved the final version of this manuscript.
